# *Pneumocystis jirovecii* Pneumonia in Patients with or without AIDS, France

**DOI:** 10.3201/eid2009.131668

**Published:** 2014-09

**Authors:** Antoine Roux, Emmanuel Canet, Sandrine Valade, Florence Gangneux-Robert, Samia Hamane, Ariane Lafabrie, Daniéle Maubon, Anne Debourgogne, Soléne Le Gal, Fréderic Dalle, Marion Leterrier, Dominique Toubas, Christelle Pomares, Anne Pauline Bellanger, Julie Bonhomme, Antoine Berry, Isabelle Durand-Joly, Denis Magne, Denis Pons, Christophe Hennequin, Eric Maury, Patricia Roux, Élie Azoulay

**Affiliations:** Centre Medico-Chirurgical FOCH, Suresnes, France (A. Roux);; Hôpital Saint-Louis, Paris, France (E. Canet, S. Valade, S. Hamane, A. Lafabrie, É. Azoulay);; Centre Hospitalier Universitaire (CHU) de Rennes, Rennes, France (F. Gangneux-Robert );; CHU de Grenoble, Grenoble, France (D. Maubon);; CHU de Nancy, Nancy, France (A. Debourgogne);; Centre Hospitalier Régional et Universitaire de Brest, Brest, France (S. Le Gal);; CHU de Dijon, Dijon, France (F. Dalle);; CHU de Nantes, Nantes, France (M. Leterrier);; CHU de Reims, Reims, France (D. Toubas);; Hôpital de l’Archet, Nice, France (C. Pomares);; CHU Jean Minjoz, Besançon, France (A.P. Bellanger);; CHU Côte de Nacre, Caen, France (J. Bonhomme);; CHU de Toulouse, Toulouse, France (A. Berry);; CHU de Lille, Lille, France (I. Durand-Joly);; Hôpital Saint-Antoine, Paris (D. Magne, C. Hennequin, P. Roux, E. Maury);; CHU Gabriel Montpied, Clermont-Ferrand, France (D. Pons)

**Keywords:** Immune suppression, HIV/AIDS, cancer, transplantation, mechanical ventilation, viruses, immunosuppressive disorder, France, fungi, Pneumocystis jirovecii, pneumonia, respiratory infections, prophylaxis

## Abstract

*Pneumocystis jirovecii* pneumonia (PCP) in patients without AIDS is increasingly common. We conducted a prospective cohort study of consecutive patients with proven PCP; of 544 patients, 223 (41%) had AIDS (AIDS patients) and 321 (59%) had other immunosuppressive disorders (non-AIDS patients). Fewer AIDS than non-AIDS patients required intensive care or ventilation, and the rate of hospital deaths—17.4% overall—was significantly lower for AIDS versus non-AIDS patients (4% vs. 27%; p<0.0001). Multivariable analysis showed the odds of hospital death increased with older age, receipt of allogeneic bone marrow transplant, immediate use of oxygen, need for mechanical ventilation, and longer time to treatment; HIV-positive status or receipt of a solid organ transplant decreased odds for death. PCP is more often fatal in non-AIDS patients, but time to diagnosis affects survival and is longer for non-AIDS patients. Clinicians must maintain a high index of suspicion for PCP in immunocompromised patients who do not have AIDS.

## Abstract 2

Immunosuppressed patients without AIDS had longer time to treatment and a higher rate of death than did patients with AIDS.

*Pneumocystis jirovecii* pneumonia (PCP), caused by the fungus *P.* *jirovecii* (formerly *P. carinii*), is a life-threatening, opportunistic infection that is often the AIDS-defining illness in patients with HIV infection. Consequently, PCP has been extensively studied as a manifestation of the AIDS epidemic. However, in high-resource countries, the decrease in the prevalence of AIDS and the use of highly active antiretroviral therapy and routine primary PCP prophylaxis have diminished the number of patients with HIV-related PCP (*1*,*2*). At the same time, PCP has emerged as a concern in patients with non–HIV-related immune deficiencies. PCP has become more common among these patients as a result of treatment changes such as increasing use of immunosuppressive agents to treat malignancies, autoimmune diseases, and inflammatory diseases, as well as an increase in the number of solid organ transplants (SOTs) (*3*,*4*). Thus, patients who have hematologic or solid-organ malignancies or autoimmune or chronic inflammatory diseases, or those who have received an SOT or hematopoietic stem cell transplant (HSCT), are at high risk for PCP (*5*–*8*).

Whether these changes in PCP epidemiology have affected the clinical manifestation and outcome of the disease remains unclear. As early as 1989, a biological study that compared PCP in patients with and without AIDS found significant differences in fungi counts and lung inflammation (*9*). Another study evaluated clinical manifestations and outcomes of PCP in patients without AIDS seen during 1980–1993 (*10*) but did not include a comparison with AIDS patients. In a study comparing PCP in AIDS and non-AIDS patients in Basel, Switzerland, during 1982–1998, non-AIDS patients more often required intensive care and mechanical ventilation, although rates of death were not significantly different for the 2 groups (*11*). The Switzerland study and another comparison of AIDS and non-AIDS patients with PCP published in 1984 (*12*) found that symptom duration was longer and oxygen tension needs higher for AIDS patients. 

These studies suggest that differences in PCP pathogenesis and influences of the underlying disease or treatment may affect the expression of PCP. However, recent data are lacking on the differences between clinical features and outcomes of PCP in AIDS and non-AIDS patients. Clinicians need this information to help identify patients who require prophylaxis and to enable early diagnosis of PCP at a stage when treatment is most likely to be effective. To obtain data on manifestations and outcomes of PCP in recent years and to identify risk factors for death, we performed a prospective, multicenter, observational study of consecutive patients with confirmed PCP admitted to 17 hospitals in France during 2007–2010.

## Materials and Methods

### Patients and Management

The appropriate ethics committee approved this study; informed consent was not required because of the observational design. For the study, the head mycologist at each of 17 university-affiliated hospitals in France prospectively included consecutive patients with confirmed PCP who were admitted during January 1, 2007–December 31, 2010. We defined confirmed PCP as a positive result for *Pneumocystis jirovecii* by Gomori-Grocott or toluidine blue stain or positive immunofluorescence test results (*4*) for a bronchoalveolar lavage (BAL) fluid or induced sputum specimen. Induced sputum testing or bronchoscopy with BAL was performed at the discretion of the clinicians by using previously described procedures (*13*,*14*). We did not include patients for whom only PCR results were positive.

For all included patients, chest radiographs were obtained; computed tomography (CT) scans were performed when deemed necessary by clinicians. Bilateral interstitial or alveolointerstitial opacities on chest radiographs and diffuse ground-glass opacities on CT images were considered typical findings for PCP. Septal lines and centrolobular nodules were also interpreted to support a diagnosis of PCP. Focal consolidation, pleural effusion, subpleural nodules, and cavitation were considered atypical findings. Criteria for microbiologically documented pneumonia were as follows: clinical symptoms of pneumonia; pulmonary infiltrates; and >1 positive noncolonizing microbiological sample (i.e., blood culture, tracheal aspirate, sputa examination, BAL, protected sample, or pleural fluid). Urine antigens (*Streptococcus pneumoniae* and *Legionella pneumophila*) were included in routine testing for the microbiological documentation of pneumonia. When the microbiological data were negative but the patient had symptoms of pneumonia and pulmonary infiltrates, the case was classified as clinically documented pneumonia (*13*).

### Data Collection

Data were collected prospectively. Steroid treatment was either high dose (>1 mg/kg for >1 mo) or long term (>3 mo at any dose) (*3*,*14*). Mycologists and study investigators obtained missing and follow-up data by reviewing patients’ medical charts and by interviewing the specialists who provided usual care to the patients. Bacterial, viral, fungal, and parasitic infections were diagnosed on the basis of criteria reported elsewhere (*13*). Information on PCP prophylaxis was recorded; trimethoprim-sulfamethoxazole (TMP-SMX), aerosolized pentamidine (1×/mo), dapsone, and atovaquone were classified as effective prophylaxis options (*3*,*4*). Information on treatments used for PCP and the time from admission to treatment initiation were recorded; TMP-SMX, pentamidine, atovaquone, dapsone, and clindamycin-primaquine were considered acceptable options for PCP treatment (*4*). Shock was defined as persistent hypotension despite appropriate fluid load, requiring treatment with a vasopressive drug.

Steroids as adjunctive therapy were used on the basis of standard protocol recommendations for patients with AIDS at a dose that depended on patient location (e.g., medical unit). In deeply hypoxemic, critically ill patients, steroids were implemented at the dosage of 240 mg/day for 3 days, then at 1 mg/kg/day for 7 days, followed by tapering doses to be stopped before day 21 (*15*,*16*). In patients who were less critically ill, the dose was 1 mg/kg/day followed by a tapering dose after day 7 to be stopped before day 21. Similar protocols were used for AIDS and non-AIDS patients.

### Statistical Analyses

The variables in the dataset are described or summarized by using either median and interquartile range or number and proportion of the total (%). Categorical variables were compared by using the Fisher exact test and continuous variables by using the nonparametric Wilcoxon test or Mann-Whitney test for pairwise comparisons. All tests were 2-sided, and p<0.05 was considered statistically significant. Kaplan-Meier curves and log-rank tests were used to compare hospital death rates between groups of patients. Logistic regression analysis was used to identify variables significantly associated with hospital deaths by estimating the odds ratios (OR) with 95% CIs. Variables yielding p values <0.20 in the univariable analyses were entered into a multivariable logistic regression model with stepwise variable selection using an automatic procedure based on the Akaike Information Criterion, with hospital deaths as the variable of interest. The co-variates were entered into the model with a p value cutoff for removal of 0.1. Co-linearity and interactions were tested. For the multivariable analysis, missing data were handled by using multiple imputation with chained equations. The imputed missing data focused on 3 variables: oxygen saturation on admission (35 patients), time to treatment (17 patients), and time from respiratory symptom onset to diagnosis (11 patients). The Hosmer-Lemeshow test was used to check goodness-of-fit of the logistic regression model.

## Results

### Causes of Immunodeficiency

**Figure 1:**
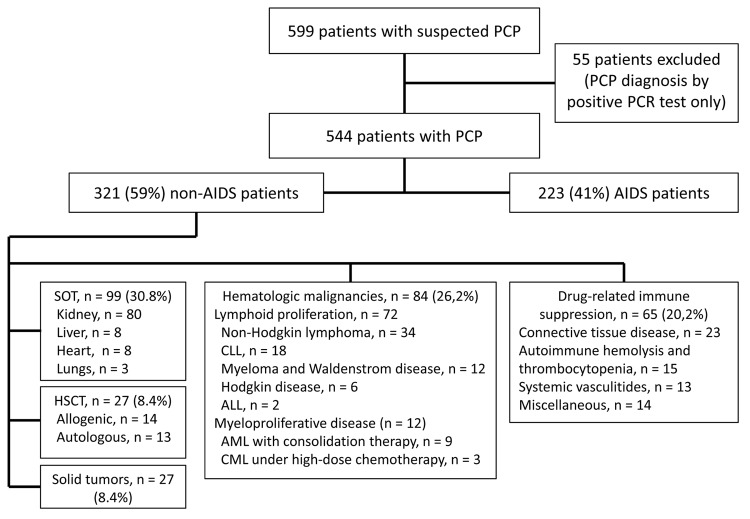
Flowchart of selection of patients with *Pneumocystis jirovecii* pneumonia (PCP) for study and underlying conditions among non-AIDS patients, France, January 1, 2007–December 31, 2010. Miscellaneous conditions: inflammatory diseases or automimmune (n = 4); common variable immunodeficiency (n = 2); focal segmental glomerulosclerosis (n = 2); sarcoidosis (n = 1); steroid-dependent asthma (n = 1); idiopathic pulmonary fibrosis (n = 1); acute alcoholic hepatitis (n = 3). ALL, acute lymphoid leukemia; AML, acute myeloid leukemia; CLL, chronic lymphoid leukemia; CML, chronic myeloid leukemia; HSCT, hematopoietic stem cell transplant; SOT, solid organ transplant.

We included 544 patients in the study. Median age was 51 (interquartile range 40–62) years, and 370 (68%) were men A total of 223 (41%) patients had AIDS and 321 (59%) had other immunosuppressive conditions (Figure 1). Among patients with AIDS, PCP was the first manifestation of AIDS for 105 (44.8%); only 4 (5.0%) had CD4 lymphocyte counts >200 cells/mm^3^. As shown in Table 1, the main causes of immunodeficiency in the non-AIDS patients were SOT (n = 99, 30.8%), chiefly of a kidney (80/99); and hematologic malignancies (n = 84, 26.2%), chiefly lymphoproliferative diseases (72/84). Twenty-seven (8.4%) patients were HSCT recipients (14 allogeneic, 13 autologous); 65 (20.2%) had autoimmune or chronic inflammatory disease; and 46 (14.3%) had solid-organ malignancies.

**Table 1:** Clinical characteristics of 544 patients with and without AIDS at diagnosis with PCP, France, January 1, 2007–December 31, 2010*

Characteristic	AIDS patients, n = 223	Non-AIDS patients, n = 321	p value
Clinical features			
Prophylaxis prescribed†	3 (1)	12 (4)	0.06
Temperature >38°C	165 (74)	263 (82)	0.05
Days from constitutional symptom onset to diagnosis, median (IQR)	30 (14–60)	7 (2–15)	<0.0001
Shock	5 (2.2)	23 (7)	0.01
Respiratory symptoms			
Cough	170 (76.2)	173 (54)	<0.0001
Dyspnea	176 (79)	234 (73)	0.10
Days from respiratory symptom onset to diagnosis, median (IQR)	21 (7–30)	5 (1–15)	<0.0001
Laboratory test results			
SpO_2_, median (IQR)	95 (90–97)	91 (86–96)	0.003
Lymphocyte count, cells/mm^3^, median (IQR)	802 (499–1,200)	500 (278–880)	0.0004
CD4+ T-cell count, cells/mm^3^, median (IQR)	167 (89–342)	32 (12–75)	<0.0001
C-reactive protein	48 (17–128)	120 (59–210)	<0.0001
Radiologic findings			
Chest radiograph results typical for PCP	183 (82)	247 (77)	0.23
Chest radiograph results atypical for PCP‡	31 (14)	48 (15)	0.66
Pneumothorax	7 (3.1)	7 (2.2)	0.50
Chest radiograph results unremarkable	9 (4)	26 (8)	0.34
Atypical computed tomography scan pattern§	15 (14)	22 (14)	0.47

*Values are no. (%) patients except as indicated. Data were missing for CD4+ T-cell count (372 patients), C-reactive protein (274 patients), and CT scan pattern (265 patients). PCP, *Pneumocystis jirovecii* pneumonia; IQR, interquartile range. †Adherence to prescribed prophylaxis was unknown. Among already known HIV+ patients without prophylaxis and with available CD4+ T cell count (n = 74), median CD4+ T cell count was 33 cells/mm^3^ (range 12–85). ‡Defined as focal interstitial or alveolar consolidation. §Defined as subpleural nodules, focal condensation, cavitation, or marked pleural effusion.

At the time of PCP diagnosis, high-dose or long-term steroid therapy was the most common immunosuppressive treatment for non-AIDS patients: 88% (n = 63) for those with SOTs, 85% (n = 71) for those with hematologic malignancies, 100% (n = 13) for those with HSCTs, 91% (n= 41) for those with solid-organ malignancies, and 89% (n = 52) for those with autoimmune or chronic inflammatory disease. SOT recipients were also receiving anticalcineurin agents (n = 59, 82%), purine inhibitors (n = 56, 77%), rituximab (n = 1, 6%), mechanistic target of rapamycin inhibitors (n = 6, 8%), tumor necrosis factor–α antagonists (n = 3, 4%), or intravenous immunoglobulins (n = 1, 1%). Patients with hematological malignancies also had received rituximab (n = 29, 35%) and fludarabin (n = 8, 9.5%). All HSCT patients were receiving anticalcineurin agents. Patients with autoimmune or chronic inflammatory diseases were receiving methotrexate (n = 14, 21.5%), rituximab (n = 6, 9.2%), tumor necrosis factor–α antagonists (n = 6, 9.2%), anticalcineurin agents (n = 5, 7.7%), or purine inhibitors (n = 4, 6.1%). (Note that values are approximate; data are missing among each subpopulation.)

### Clinical Manifestations of PCP

The clinical manifestations of PCP in AIDS and non-AIDS patients are shown in Table 2 and Figure 1. Overall, 96% of patients were not receiving PCP prophylaxis. The median time from the onset of respiratory symptoms to PCP diagnosis was significantly shorter for non-AIDS patients (5 [range 1–15] days) than for AIDS patients (21 [7–30] days) (p<0.0001). However, as shown in Tables 1 and 2, hypoxemia was more severe in non-AIDS patients, who required higher oxygen flow rates (4 [range 3–5] L/min) than did AIDS patients (2 [1.3–3] L/min). Non-AIDS patients also more often required intensive care and noninvasive or invasive ventilation. Shock was more common in non-AIDS patients and was significantly associated with pulmonary microbial co-infection (OR 3.09 [95% CI 1.44–6.68]; p = 0.004), in agreement with the presence of microbiologically documented pneumonia (*13*) in half of the patients with shock. CT scans were obtained for 279 patients; typical findings were seen in 37 patients, of whom 29 were believed to have pulmonary bacterial microbial co-infection and 8 microbial co-infections with a second opportunistic microorganism.

**Table 2:** Clinical management of 544 AIDS and non-AIDS patients after diagnosis with PCP, France, January 1, 2007–December 31, 2010*

Characteristic	AIDS patients, n = 223	Non-AIDS patients, n = 321	p value
Days from admission to treatment initiation, median (IQR)	1 (0–2)	2 (0–6)	<0.0001
Intensive care admission	65 (35)	134 (50)	0.0015
Immediate oxygen needed	87 (49)	160 (69)	<0.0001
Oxygen flow rate, L/min, mean (95% CI)	2 (1.3–2.8)	3.8 (2.8–4.8)	0.015
Mechanical ventilation			
Noninvasive needed	17 (8)	50 (16)	0.0053
Noninvasive failed	16 (8)	46 (15)	0.013
Invasive needed	25 (11.0)	98 (30.5)	<0.0001
Hospital deaths	8 (4)	75 (27)	<0.0001


*Values are no. (%) patients except as indicated. PCP, *Pneumocystis jirovecii* pneumonia; IQR, interquartile range.

### Diagnosis and Treatment of PCP

A BAL sample was diagnostic for 87% and 97% of AIDS and non-AIDS patients, respectively (p = 0.0003). Overall, microbial co-infection as previously defined (*13*) was suspected or confirmed for 169 (31%) patients, including 92 (16.9%) with bacterial infection, 65 (6.6%) with viral infection, and 38 (6.9%) with fungal infection. For 32 (5.8%) patients, >2 microbial co-infections were identified; the most frequent site of microbial co-infection was the lung (Technical Appendix Table). Univariable analysis showed an association between microbial co-infection and death (OR 2.29, 95% CI 1.41–3.71) (Technical Appendix Table), but multivariable analysis did not confirm this finding (OR 1.99, 95% CI 0.91–4.33; p = 0.09).

Time from hospital admission to the start of treatment for PCP was longer for non-AIDS patients than for AIDS patients (2 [range 0–6]) days vs. 1 [0–2] days; p<0.0001). TMP-SMX was the first-line anti-PCP agent used for 97% of patients. However, patients with AIDS more often required a switch to second-line therapy than did non-AIDS patients (25.5% vs. 7%; p<0.0001); this change was most often made because of allergic reactions or hepatic or renal toxicity. Adjunctive steroid therapy was used in 40.4% of patients overall and in a significantly higher proportion of AIDS than in non-AIDS patients (55% vs. 43%; p = 0.01).

### Risk Factors for Death

Hospital death data were available for 478 (88%) patients, and the percentage of hospital deaths was significantly higher for non-AIDS than AIDS patients (27% vs. 4%; p<0.0001). For non-AIDS patients, death rates varied by cause of immunodeficiency, from a low of 3.75% for kidney transplant recipients to a high of 43% for allogeneic HSCT recipients. Invasive mechanical ventilation was used in 28% and 43% of patients in these 2 groups, respectively (p<0.0001).

Of 7 variables independently associated with hospital death by multivariable analysis, 2 were associated with lower death rates: AIDS diagnosis (OR 0.33, 95% CI 0.12–0.92) and receipt of a SOT (OR 0.08, 95% CI 0.02–0.31) (Table 3). Five variables were associated with increased death rates: older age (OR 1.04/additional year, 95% CI 1.02–1.06); receipt of a HSCT (OR 8.6, 95% CI 1.40–53.02); need for oxygen on admission (OR 4.06, 95% CI 1.44–11.5); need for invasive mechanical ventilation (OR 16.70, 95% CI 7.25–38.47); and longer time from admission to initiation of PCP treatment (OR 1.11/additional day, 95% CI 1.04–1.18).

**Table 3:** Multivariate analysis of independent predictors of hospital death for AIDS and non-AIDS patients with PCP, France, January 1, 2007–December 31, 2010*

Variable	Odds ratio (95% CI)
HIV infection	0.33 (0.12–0.92)
Solid organ transplant	0.08 (0.02–0.31)
Age, per additional year	1.04 (1.02–1.06)
Allogeneic HSCT	8.6 (1.40–53.02)
Need for immediate oxygen therapy	4.06 (1.44–11.5)
Need for intubation and mechanical ventilation	16.70 (7.25–38.47)
Time to PCP treatment, per additional day	1.11 (1.04–1.18)

*Variables from Tables 1 and 2 were introduced into the multivariate model based on their association with hospital mortality by bivariate analysis with p values <0.20. Higher numbers indicate increased odds, with 1 as the cutoff point (i.e., values <1.00 indicate decreased risk, >1.00 increased risk). Goodness of fit (Hosmer-Lemeshow test) was 0.61. PCP, *Pneumocystis jirovecii* pneumonia; HSCT, hematopoietic stem cell transplant.

**Figure 2:**
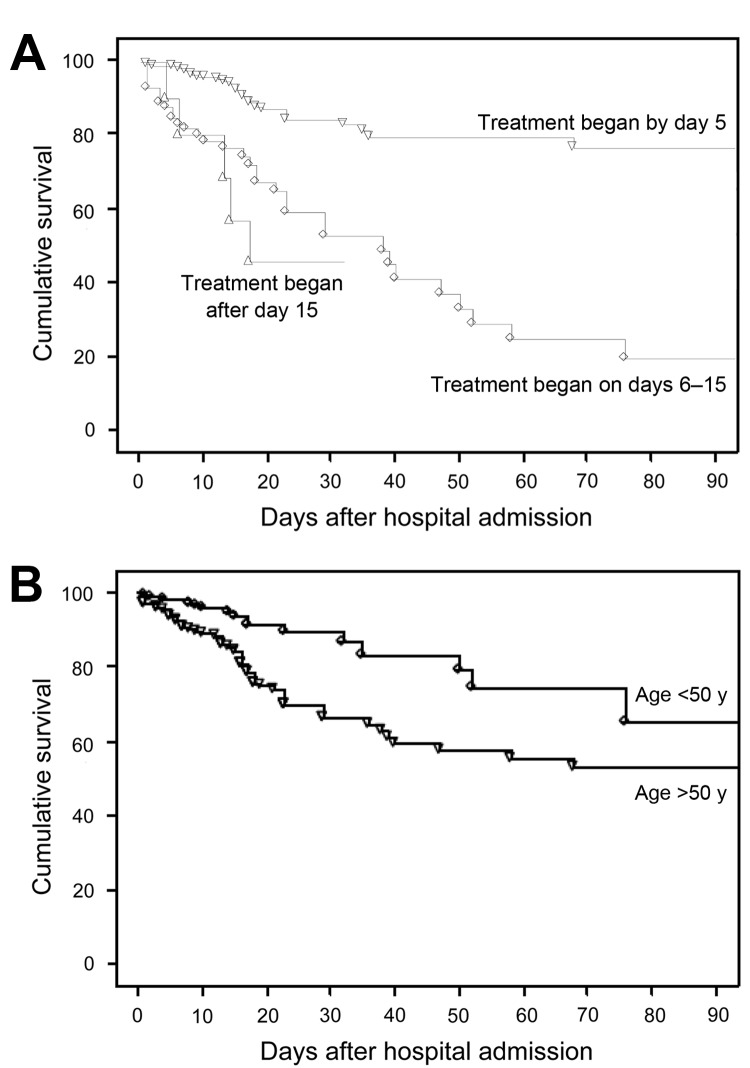
Survival in 544 patients with *Pneumocystis jirovecii* pneumonia by A) number of days from admission to treatment initiation and B) patient age, France, January 1, 2007–December 31, 2010. p<0.0001 by log-rank test for both comparisons.

Improved cumulative survival was significantly associated with underlying condition (p<0.0001 for AIDS vs. non-AIDS comparison; Figure 2). Shorter time from admission to treatment initiation was also associated with improved cumulative survival (Figure 2).

## Discussion

This multicenter, prospective study describes the current picture of PCP in immunocompromised patients with or without AIDS in a high-resource country. In this cohort, AIDS-related PCP was less common than was PCP associated with other types of immunosuppression. Our findings confirm several differences between AIDS and non-AIDS patients in clinical presentation and outcomes related to PCP, as described by Kovacs et al. (*12*). The progression of PCP was faster for non-AIDS patients; these patients had a significantly shorter time from onset of symptoms to diagnosis but still experienced a faster progression of illness, including more severe hypoxemia, greater need for intensive care and invasive mechanical ventilation, higher prevalence of shock, and a longer time to PCP treatment initiation. Death rates were also significantly higher for non-AIDS patients, and one of the variables independently associated with death was longer time to PCP treatment initiation for non-AIDS patients.

This study offers several contributions toward the development of PCP prophylaxis guidelines for specific at-risk groups. One of the reasons for the lower proportion of AIDS patients than of non-AIDS patients in this study population could have been the widespread use of highly active antiretroviral therapy and PCP prophylaxis among AIDS patients (*1*,*2*). However, among patients with AIDS in our study, only 3 (2.7%) were receiving PCP prophylaxis; for 100 (44.8%) patients, the PCP diagnosis was the reason for the AIDS diagnosis.

In our study, PCP treatment was started later after admission in non-AIDS patients than in AIDS patients, and longer time to treatment independently predicted odds for death, which is in agreement with findings of an earlier study (*12*). Longer time to treatment was the only predictor of death in our study that could be mitigated. Treatment initiation differed by only 1 day for AIDS versus non-AIDS patients, yet this difference was associated with reduced death rates for AIDS patients. Therefore, because routine implementation of PCP treatment on admission may be associated with higher survival, clinicians should implement treatment as soon as the diagnosis is suspected, without waiting the 2 days required to confirm the diagnosis.

Our findings indicate that PCP prophylaxis could improve outcomes for high-risk patients without AIDS. Among non-AIDS patients in this study, 99 (30.8%) were SOT recipients, a population for which recent guidelines recommend PCP prophylaxis for 6–12 months, a period that might be extended on the basis of level of immunosuppression and immunosuppressive drug requirements (*18*,*19*). Despite this recommendation, however, recent studies suggest that 1 month of prophylaxis would be sufficient for kidney transplant patients (*20*). Given the high rate of death in our cohort, this conclusion should be challenged. Maintaining a high index of suspicion for PCP in immunocompromised patients appears to be of the utmost importance. In addition, as with AIDS patients, every effort should be made to ensure compliance with PCP prophylaxis in non-AIDS patients. Non-AIDS patients may be less aware than AIDS patients that they are at risk for PCP. This point may be of particular relevance, as the course of PCP was significantly more acute in non-AIDS patients, with a median symptom duration of 5 (range 1–15) days compared with 21 (7–30) days for AIDS patients (p<0.0001). Educating non-AIDS patients about PCP might result in earlier medical evaluation and hospital admission and, consequently, in shorter lengths of time to PCP diagnosis and treatment. In addition, development of rapid and minimally invasive diagnostic tests could improve the early diagnosis and treatment of PCP (*21*).

We found marked differences in death rates across patient groups. Deaths were lowest for AIDS patients and highest for HSCT recipients, and rates in our study were consistent with earlier data (*6*,*12*). Microbial co-infection rates in our study were also in agreement with earlier data (*22*). More than one fourth of our patients overall had microbial co-infection, which indicates a need for comprehensive diagnostic investigations in patients with PCP and for routine broad-spectrum antimicrobial drug therapy when findings are atypical for PCP.

Adjunctive steroid therapy has been proven to increase survival in AIDS patients with severe PCP (*23*,*24*), but 2 small retrospective studies found that adjunctive steroids had no effect on survival for non-AIDS patients (*17*,*25*). In our study, although non-AIDS patients had more severe hypoxemia and more often required invasive mechanical ventilation, they received adjunctive steroid therapy significantly less often than did AIDS patients.

Our study has several limitations. First, the patients were recruited at university hospitals, which may have influenced the distribution of risk factors for PCP. However, most of these risk factors are associated with diseases that require management in university hospitals. Our data obtained for consecutive patients from 17 centers are probably representative of PCP in other countries where optimal AIDS treatment and critical care are widely available. In addition, the diagnosis and therapeutic management of these patients was not standardized, and variations in testing and treatment strategies may have affected outcomes and determinants of death. Moreover, the diagnostic strategy may have been different for AIDS versus non-AIDS patients, as well as for critically ill patients versus non–critically ill patients; these differences could have resulted in different proportions of patients with documented microbial superinfections. However, our objective was to describe all PCP patients seen during the past few years, and we found no effect of the center at which a patient was treated on mortality rates (data not shown). This study also included only episodes of PCP for which a patient was hospitalized. AIDS patients with mild PCP episodes could be treated as outpatients, and the prognosis and characteristics for these patients may vary substantially from those of our study population. Last, we included only PCP cases proven by tinctorial or immunofluorescence staining. PCP may be present in some patients who have positive PCR test results for *P. jirovecii* but for whom stain results are negative or unavailable (*26*). However, because isolated PCR test positivity can indicate colonization and not infection (*27*), we confined our study to cases of confirmed infection to maximize the validity of our data.

In summary, PCP occurs in patients with a range of conditions associated with immunosuppression. For AIDS patients, efforts should focus on improving the early detection of HIV infection and adherence to PCP prophylaxis. For non-AIDS patients, guidelines regarding PCP risk evaluation and prophylaxis are needed. We found higher death rates and longer time from hospital admission to initiation of PCP treatment for non-AIDS patients. Clinicians must maintain a high index of suspicion for PCP in immunocompromised patients who do not have AIDS, and these patients should be educated about the early symptoms that can indicate PCP. Treatment should be implemented early in high-risk patients, even before appropriate diagnostic tests are completed.

Technical Appendix Co-infections among 544 *Pneumocystis jirovecii* pneumonia patients with and without AIDS and survival for those with versus without co-infections, France, January 1, 2007–December 31, 2010.
