# Supplementary material for: Pneumocystis jirovecii Pneumonia in Patients with or without AIDS, France
Source: Emerg Infect Dis. 2014 Sep;20(9):1490–7. doi: 10.3201/eid2009.131668 (PMC4178412; doi:10.3201/eid2009.131668)
Supplement: Technical Appendix — Co-infections among 544 Pneumocystis jirovecii pneumonia patients with and without AIDS and survival for those with versus without co-infections, France, January 1, 2007–December 31, 2010. [file 13-1668-Techapp-s1.pdf]

Article DOI: http://dx.doi.org/10.3201/eid2009.131668

Pneumocystis jirovecii Pneumonia in Patients with or without AIDS, France

Technical Appendix

Technical Appendix Table. Description of the cases of microbial co-infections in 544 patients with and without AIDS, France, January 1, 2007–December 31, 2010*

Co-infection	All, n = 544	AIDS, n = 223	Non-AIDS, n = 331	p value
≥1 microbial co-infection	169 (31)	68 (30.5)	101 (30.5)	0.99
≥2 microbial co-infections	32 (5.8)	12 (5.3)	20 (6)	0.83
Pathogen				
Virus	65 (11.9)	30 (13.4)	35 (10.5)	0.34
CMV	44	21	23	
HSV	18	7	11	
Influenza	2	1	1	
RSV	1	1	0	
Bacteria	92 (16.9)	41 (18.3)	37 (11.1)	0.018
Pneumococcus	12	6	6	
Enterococcus sp.	4	3	1	
Streptococcus sp.	4	2	2	
Haemophilus or *moraxella catharallis*	8	6	2	
Staphylococcus aureus	13	11	2	
Other Staphylococcus spp.	5	1	4	
Pseudomonas	17	6	11	
Enterobacteria	13	4	9	
Mycobacterium tuberculosis	2	2	0	
Others	14			
Fungus	38 (6.9)	10 (4)	28 (8)	0.08
Aspergillus sp.	18	0	18	
Candida sp.	20	10	10	
Cryptococcus neoformans	2	2	0	
Parasite	6 (1.1)	6 (2.6)	0 (0)	0.0041
Cryptosporidum sp.	1	1	0	
Toxoplasma gondii	1	1	0	
Schistosoma sp.	1	1	0	
Isospora belli	1	1	0	

*Values are no. (%) patients. p values by χ^2 test. CMV, cytomegalovirus; HSV, herpes simplex virus; RSV, respiratory syncytial virus.

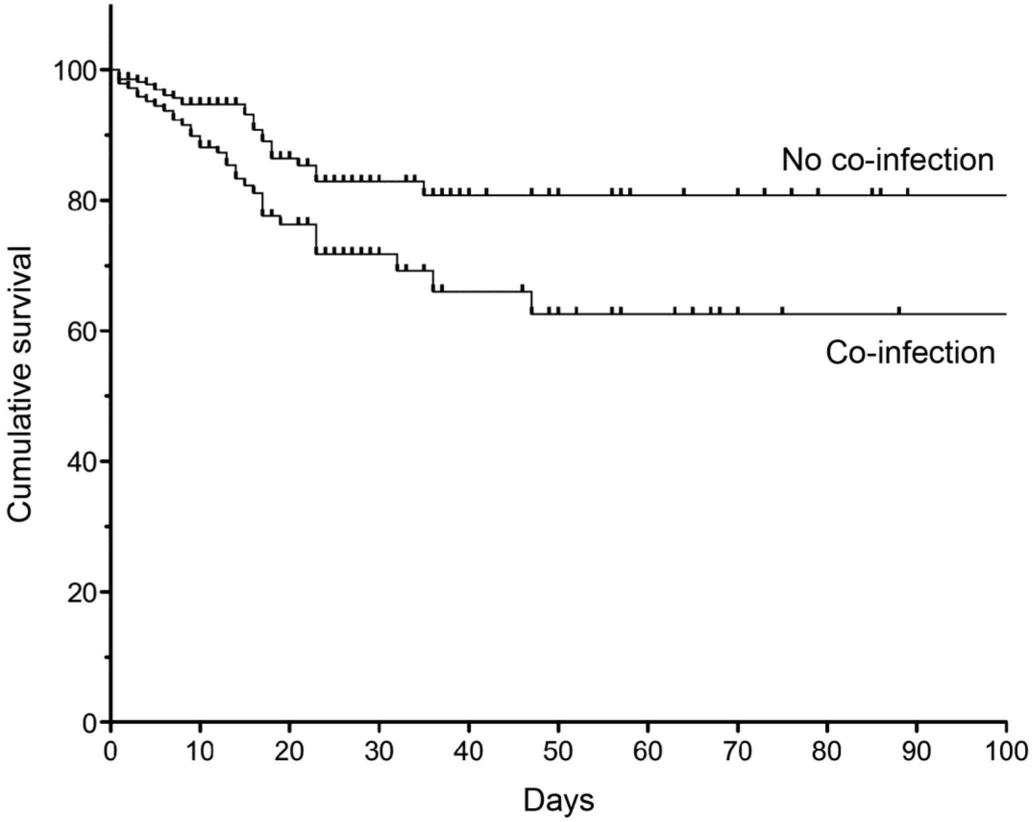

Technical Appendix Figure. Survival rates for 544 *Pneumocystis jiroveci* pneumonia patients with and without co-infections, France, January 1, 2007–December 31, 2010. Days, days after hospital admission. p = 0.0031 by log-rank test.
